# Electronic Noses for Environmental Monitoring Applications

**DOI:** 10.3390/s141119979

**Published:** 2014-10-24

**Authors:** Laura Capelli, Selena Sironi, Renato Del Rosso

**Affiliations:** Politecnico di Milano, Department of Chemistry, Materials and Chemical Engineering “Giulio Natta”, Piazza Leonardo da Vinci 32, Milano 20133, Italy; E-Mails: selena.sironi@polimi.it (S.S.); renato.delrosso@polimi.it (R.D.R.)

**Keywords:** environment, odor, dynamic olfactometry, chemical analysis, training, sensors, classification, discrimination, quantification, odor concentration

## Abstract

Electronic nose applications in environmental monitoring are nowadays of great interest, because of the instruments' proven capability of recognizing and discriminating between a variety of different gases and odors using just a small number of sensors. Such applications in the environmental field include analysis of parameters relating to environmental quality, process control, and verification of efficiency of odor control systems. This article reviews the findings of recent scientific studies in this field, with particular focus on the abovementioned applications. In general, these studies prove that electronic noses are mostly suitable for the different applications reported, especially if the instruments are specifically developed and fine-tuned. As a general rule, literature studies also discuss the critical aspects connected with the different possible uses, as well as research regarding the development of effective solutions. However, currently the main limit to the diffusion of electronic noses as environmental monitoring tools is their complexity and the lack of specific regulation for their standardization, as their use entails a large number of degrees of freedom, regarding for instance the training and the data processing procedures.

## 1. Introduction

The concept of the electronic nose as a tool made up of sensors used to classify odors was introduced for the first time by Persaud and Dodd in 1982 [1]. In the course of their experiments, these two researchers set themselves the goal of creating a tool capable of emulating the mammalian olfactory system by recognizing different odors and giving repeatable responses. Specifically, the electronic nose they developed comprised: (I) a matrix of sensors to simulate the receptors of the human olfactory system; (II) a data processing unit that would perform the same function as the olfactory bulb; (III) a pattern recognition system that would recognize the olfactory patterns of the substance being tested, a function performed by the brain in the human olfactory system [2].

Because of the electronic nose's capability to discriminate and recognize a variety of different gases and odors using just a small number of sensors, and the first promising results obtained from research in this field, there was huge interest in the subject in the science community and over the course of time this led to a host of research projects focusing on electronic nose applications in various different areas, including, for instance, the medical and diagnostic [3–5], food [6–8] and cosmetics [9] sectors.

Research into the potential use of electronic noses in the environmental sector was also undertaken [10]. This, in fact, is the area featuring the most diverse applications, arising out of a broad range of different requirements, such as, for example, the need for a reliable, easy-to-use tool that can provide answers in real time without the waiting times required by more traditional analytical methods (e.g., chemical analyses, dynamic olfactometry) [11–13].

Although electronic noses have been widely used in both the medical and food sectors since the very first years after Persaud and Dodd published the results of their work, implementation in the environmental sectors proved to be more difficult, mostly as a result of problems related with the sensors, especially with their sensitivity and reliability. More in detail, the greatest difficulties have been encountered in field applications as the most commonly-used sensors are sensitive to variations in atmospheric conditions (*i.e.*, temperature and humidity) [14,15] and specific research is thus required to find a solution to this thorny issue before electronic noses can be used to produce reliable results in outdoor environments [16]. Furthermore, time drift in the sensors used means periodic re-calibration is necessary and the poor sensitivity of certain sensors to the presence of a variety of compounds are both problems that need to be resolved to make the use of electronic noses in environmental field applications possible [17,18]. Lastly, because of the huge variety of possible field applications, new tools have to be specifically honed for each application [19], to ensure they meet the specific requirements of that application.

In environmental field applications, electronic noses are principally used to analyze air. However, they can also be used for the evaluation of water. In these instances, the characteristics of the water can be detected by using an electronic nose in the head space above the water or using instruments with a matrix of liquid sensors that work in a way similar to human taste sensors with the result that they are commonly known as “electronic tongues” [20–25].

Electronic nose applications in environmental monitoring can be divided into the following main categories: (I) analysis of parameters relating to air quality; (II) analysis of parameters relating to water quality; (III) process control; (IV) verification of efficiency of odor control systems.

In each of these applications, the electronic nose can be used either as a replacement or add-on to other more traditional, well-established analytical methods. For instance, an opportunely-calibrated electronic nose has been proposed to evaluate BOD5 concentrations in waters downstream from effluent treatment plants as this would be far speedier than the existing tests which involve quite long waiting times [26]. Furthermore, in the area of air quality monitoring, electronic noses can be used as an alternative to gas-chromatography in evaluating the presence and concentration of various pollutants [27–29]. Finally, electronic noses can also be used as real-time process control tools, instead of costly analyses that often require long waiting times. As an example, the detection of unwanted anaerobic processes in a composting plant could be achieved combining temperature, pH, oxygen level and microbiological analysis measurements. As an alternative, similar information could be obtained by using an opportunely trained electronic nose to monitor the composting process, whereby the use of electronic noses may be cheaper, less complex [30,31] and still quite descriptive too.

Besides representing an alternative or a complementary method to analytical methods, electronic noses are also the only instruments capable of evaluating and classifying odors [32]. Classical analytical methods (*i.e.*, GC-MS) can determine the chemical composition of a gaseous mixture but the latter cannot be generally correlated to the odor concentration of the mixture, particularly in the case of complex multi-component mixes [33].

For this reason, opportune sensorial methods are currently used to quantify odor. These include dynamic olfactometry [34], which can measure so-called odor concentration which in ou_E_/m^3^ (odor units per m^3^), which represents the number of dilutions with neutral air required to bring an odorous sample to its olfactory threshold concentration.

There are also other sensorial methods, which allow hedonic tone, odor intensity and odor quality to be evaluated too. The drawback of sensorial analyses is that they are generally complex, costly and difficult to execute in real time. For these reasons, in some cases it may be preferable to use electronic noses to replace other analytical methods in the environmental field. In addition to this, they may be the only currently available method of simultaneously both evaluating the odor concentration of the analyzed air and classifying it, by attributing to it a specific olfactory class and thereby recognizing the provenance of the odor [35].

For all these reasons, several different studies have been conducted in recent times with the aim of evaluating the possibility of applying electronic nose technology to environmental applications. This article reviews the findings of those studies with particular focus on the proposed applications.

## 2. Electronic Noses

### 2.1. General

The aim of this section is to provide some general and “rough” information about electronic noses in order to make the following literature discussion more comprehensible. It is not the aim of this paper to focus on the details of electronic nose technology, which is the object of other specific review papers or handbooks [7,36,37]. An electronic nose is a device that uses an array of non- or partially-specific sensors, which can distinguish between different odors.

As reported by Gardner and Bartlett [38], an electronic nose consists of: (I) a sample delivery system that transfers air to be analyzed from the surrounding environment inside the instrument; (II) a chamber containing the sensors; (III) a signal processing system and, lastly; (IV) specific recognition and classification software.

The electronic nose does not recognize the individual odor-generating compounds, but rather provides an olfactory signature (fingerprint) of the analyzed air. To do this, the instrument must be trained, *i.e.*, it must be provided with a database of olfactory fingerprints relating to the odors to which it may be exposed to during the analysis [39]. That database is put together by analyzing air samples with known olfactory qualities at different odor concentration and thus defining the olfactory classes (odor types) to be recognized [40].

### 2.2. Sample Delivery and Capturing Systems

Depending on the type of application involved, various types of systems have been developed and are used to deliver gas samples to the inside of the electronic nose. More specifically, the main differences between the various systems relate to the type of sample requiring analysis. With regard to the electronic noses proposed for air quality monitoring, a tube connected to a dedicated pump is generally used. This sucks in the air to be sampled and delivers it to the sensor chamber [41,42].

However, in terms of electronic noses being used to monitor water quality, the air flow sent for analysis to the instrument is generally sampled from the headspace created in enclosed chambers in which the liquid to be analyzed is placed and into which odorless air is ducted [43,44].

In the case of interstitial gas in soil or emissions from solid heaps, specific hoods or wind tunnels can be used in addition to the headspace method [45,46]. The gas mix obtained at the outlet of these sampling systems can then be conveyed to the electronic nose detection system.

### 2.3. Detection System

The sensor array and the nature of each individual sensor are the most important features of any electronic nose. The volatile compounds interact with the sensor surfaces and cause a change in certain chemical and physical properties of the latter. These variations are then converted to an electronic signal which is sent to the data processing system.

Regardless of the type of sensors used and their working temperature, it is essential to keep the temperature and humidity levels of the sensor chamber as constant as possible. These parameters can be set to optimize both the absorption of the odor molecules and the successive desorption.

The number and type of sensors in an electronic nose is generally selected on the basis of the specific application. However, it is possible to pinpoint certain characteristics common to all sensors used in environmental field applications.

First and foremost, the sensors have to be partially selective *i.e.*, sensitive to the substance of interest. Furthermore, their response has to be fast, stable, reproducible and reversible [47].

Different sensor types can be used in electronic noses. These are differentiated according to the gas detection principle, *i.e.*, the specific physical property that changes due to the interaction with the odor molecules in the gas flow.

The most known are:

- conductivity sensors, such as metal-oxide semiconductors (MOS) and conducting polymers (CP) [48–53], whose detection mechanism is based on conductivity variation that occurs when volatile compound interact with the sensor sensitive layer;
- piezoelectric sensors, such as surface acoustic wave (SAW) sensors and quartz crystal microbalances (QCM) [13,54], which reveal the presence of gases on the basis of the fact that the interaction with volatile molecules cases a change in mass of the surface of the sensitive material and thus a frequency shift.

Besides these, there are also other sensor types that can be used for gas and odor detections in electronic nose systems, which have been listed in Table 1 (adapted from Wilson and Baietto [55]) indicating the sensitive material and the detection principle, *i.e.*, the property that varies when the volatile molecules interact with the sensitive material.

In most environmental applications, the sensor array of the electronic nose is made up of sensors of the same kind [56], although some research has also been carried out into hybrid systems [57].

### 2.4. Data Processing and Pattern Recognition System

#### 2.4.1. Data Processing

Pre-processing of the data coming from the sensors is required to obtain the “olfactory pattern” of the sample. This procedure involves extracting certain significant characteristics from the sensor response curves (features) in order to produce a set of numerical data that can be processed by the recognition and classification system of the electronic nose [41,58]. Different features can be extracted and used depending on the characteristics of the electronic nose used such as, for example, the type of sensors adopted, and the stability of the responses of the latter to the reference gas, to variations in humidity and temperature levels [59].

Considering that each measurement can be represented as a point in an *n*-dimensional space, where *n* is the number obtained by multiplying the number of sensors in the electronic nose by the number of features extracted for each sensor, All the recognition systems are based on the principle that the distance between two points relating to the analysis of two samples in this *n*-dimensional space, is inversely proportional to their similarity. In other words, measurements relating to samples belonging to the same olfactory class will be represented by points close to each other in said *n*-dimensional space [60].

Before carrying out the recognition, an explorative analysis of the data obtained is usually performed. The most common technique is Principal Component Analysis (PCA). This provides a preliminary but efficient evaluation of the system's ability to discriminate between different olfactory classes. This technique helps reduce the complexity of the whole dataset by reducing the *n*-dimensional space of the features extracted by the sensor responses to a space of smaller dimensions, whilst still maintaining most of the information contained in the data set. This is possible because the information contained in the set of sensor responses is often redundant [61]. This method also makes it simpler to visualize any eventual clustering of the data [62], *i.e.*, the grouping of measurements relating to the same olfactory class.

Other methods that are reported in the literature for explorative data analysis include for instance “Polar Plot” (Star Plot) analysis [63,64] and Hierarchical Cluster Analysis (HCA) [65]. Exploratory data analysis is generally performed on data used to train electronic noses (*i.e.*, data provided to the instrument on the basis of which samples containing unknown olfactory classes will be classified).

#### 2.4.2. Pattern Recognition Systems

After pre-processing the information coming from the sensors, a set of numerical data is obtained, that can be further processed by suitable recognition and classification/quantification systems.

The methods for electronic nose data processing and classification (qualitative or quantitative) include specific algorithms, such as “K-Nearest Neighbors” (KNN) [66], “Discriminant Function Analysis” (DFA) [67–72], “Partial Last Squares Interpolation” (PLS) [73], or more complex systems, such as “Artificial Neural Networks” (ANN) [74,75] and Fuzzy Logic [59,76].

The KNN method is widely used because it is conceptually simple and relatively easy to implement [66]. For classification, the KNN method simply calculates the Euclidean distances between the measurement point of the sample to be classified and the points relating to the training data that identify the different olfactory classes. The unknown sample is attributed to the olfactory class to which most of the k points nearest to it belong [67,77]. Although very simple, this method does provide satisfactory results and some papers have reported classification accuracy values of over 90% [41,78].

On the other hand, Artificial Neural Networks (ANN) can be of different types [79], but in general they simulate the operating logic of the human brain through logical operations carried out by a number of processing units. In general, during the training phase, the “weight” values for the individual processing units (“neurons”) in the neural network are set-up in such a way as to guarantee the correct classification of the samples from known olfactory classes [62,80,81].

## 3. Electronic Nose Applications in Environmental Monitoring

The electronic nose applications in environmental monitoring found in the literature can be classified into four main categories: air quality monitoring (Section 3.1), water quality monitoring (Section 3.2), process control (Section 3.3) and pollution/odor control system efficiency verification (Section 3.4).

All the aforementioned applications involve laboratory and field research, with some focusing on the specific applicability and reliability of electronic noses, as discussed in the following sections.

### 3.1. Air Quality Monitoring

#### 3.1.1. General

Probably the most important area of environmental application for electronic noses is air quality monitoring. In fact, the need for an instrument capable of evaluating air pollution quickly, economically and continuously, has led many researchers to study how develop specific electronic noses for that specific purpose.

While on the one hand, air quality has already been related to the presence/absence of specific chemical pollutants, in recent times, this has been broadened to include odor annoyance as a form of air pollution. Therefore, aside from developing electronic noses to detect specific pollutants, many studies w carried out on the use of electronic noses applied for odor detection, classification and/or quantification. This is particularly interesting because while other better established and solid analytical techniques can be used to detect specific compounds, electronic noses are currently the only method capable of quantifying and classifying odors in real time. In fact, many studies discuss the fact that chemical analysis is not always suited to quantifying odors because of the interactions that can take place between the different molecules in an odorous mixture, in addition to the difficulty of detecting the presence of molecules responsible for olfactory perceptions, which are sometimes present at very low (ppb) concentrations [82–84].

As far as odor quantification is concerned, dynamic olfactometry is the official method used to determine the odor concentrations of gas samples [34]. This technique allows one to measure the odor concentration of emissions, but it does not provide any information regarding the type of odor. Furthermore, dynamic olfactometry is not suitable for measuring the presence of odors at the receptors, nor for evaluating odor exposure, whereby the odors are characterized by very low odor concentrations, often comparable to those typical of ambient air [85]. Additionally, this method cannot be used for continuous monitoring purposes because of its intrinsic discontinuity and the high costs associated with sampling and analysis. As a consequence, electronic noses are currently considered as the only possible tool for continuously analyzing air in order to detect the presence of odors and defining the quality and/or concentration of said odors.

#### 3.1.2. Detection and Quantification of Specific Compounds in Ambient Air

One of the first studies to evaluate the possibility of using an electronic nose to identify specific environmentally-relevant compounds was carried out in 1995 by Hodgins [10], who used an electronic nose equipped with CP sensors to analyze several different samples containing ethanol, dimethyl sulfide and diacetyl obtained from water mixtures containing concentrations of above 50 ppb. The instrument used turned out to be capable of distinguishing between the different substances tested, thereby indicating changes in the percentage of the resistance value with respect to the reference of at least 10%. Nonetheless, the study highlighted the need to evaluate establish the amount of time needed for the odorant mix to flow over the sensors to achieve a constant resistance value.

In order to establish the optimal contact time between substances and sensors, Abbas *et al.* [27] carried out a study using nitrogen oxide, methane and carbon monoxide in concentrations between 500 ppb and 2000 ppm. In this case, the responses of the MOS sensors used achieved a stable value after around 30 min of exposure to the air mixes containing those substances. That exposure time (30 min) was adopted for analyses of mixes containing different concentrations of the compounds analyzed with variations of other operating parameters such as relative humidity of the samples and external temperature. These tests highlighted how the sensor responses and, consequently, the ability of the electronic nose to recognize and quantify the substances present in the gas mixes are heavily influenced by those parameters. On the other hand, when the temperature and humidity values were kept constant, the system was capable of recognizing the various compounds tested with a good degree of accuracy.

A similar study conducted on polluting gas mixes at variable humidity and temperature levels was carried out by Ozmen and Dogan [29]. Using an electronic nose equipped with QCM sensors, they analyzed gas mixes containing acetone, chloroform and methanol at different concentrations (between 4000 and 10,000 ppm) to verify the system's ability to distinguish between the different compounds and establish their concentrations by means of PCA. The results obtained were satisfying in that the system differentiated between mixtures containing a single compound from binary ones, and the sensor responses changed quite appreciably as the concentrations of the chemical compounds varied. Nonetheless those results proved to be heavily influenced by the humidity level of the gas samples analyzed. Thus the authors highlight the need to relate the sensor responses to the humidity level in order to consider to use the instrument in an outdoor environment, where said humidity levels can fluctuate quite considerably, in order reduce interferences and obtain reliable results.

Further studies highlight another critical factor in using electronic nose to detect specific compounds, which is the interference caused by the co-presence of other compounds, even non-odorant ones. Helli *et al.* [28] conducted a study on this, using MOS sensors to detect the presence and concentration of H_2_S and NO_2_ in atmospheres containing CO_2_ and with certain levels of humidity. The concentrations of the gases studied were between 1 and 11 ppm for H_2_S and 1 and 5 ppm for NO_2_. The study showed how, by using discriminant factor analysis, the electronic nose can correctly estimate the composition of the tested mix. That said, that recognition is influenced by the presence of CO_2_ and the humidity in the mix, and is accurate only if those two parameters are known.

Negri and Reich [86] encountered no such dependence of the measurements from interfering substances. They used an electronic nose with MOS sensors to identify CO, isobuthane, methane and ethanol in atmospheres containing interfering gases. However, in their study, the system they developed was capable of correctly estimating 85% of the concentrations of the different compounds being tested with an error of less than 10%. Those were very satisfying results, even though it has to be taken into account that the concentrations of the substances analyzed were quite high (1000–5000 ppm) compared to the levels that are normally encountered in ambient air. That is why successive studies concentrate principally on recognition and quantification of volatile organic substances (VOC) at lower concentrations.

Wolfrum *et al.* [87], by using 14 MOS sensors, have obtained satisfactory results in the recognition and quantification of some VOCs, such as toluene, acetone and isopropanol, even at very low concentration levels (ppb). In order to establish a linear correlation between electronic nose responses and VOC concentration, the acquired data were pre-processed and subsequently used for quantification. This way, a linear correlation between real concentration and concentration estimated by the instrument was obtained, having a mean square error of prediction relevant to the three different compounds tested of 0.008, 0.011 and 0.026, respectively.

Another study aiming to realize an electronic nose capable of detecting the presence of VOC at concentrations below their TLV was conducted by Lee *et al.* [81]. Using an electronic nose based on MOS sensors, promising results were obtained by analyzing samples containing benzene, toluene, ethyl alcohol, methyl alcohol and acetone. However, the presence of sensor drift over time, *i.e.*, the variation of sensor responses when subject to the same substance over time, caused a reduction of the recognition reliability with time.

The problem of sensor drift over time, which leads to scarce reliability of the system after a certain time period, is also reported by Barisci *et al.* [88]. These authors reported their study on the development of an electronic nose based on CP sensors for the recognition of benzene, toluene, ethyl benzene and xylenes. By means of a PCA of the analyzed data it is possible to observe that the system is able to discriminate between the different compounds being tested and their different concentrations, comprised between 1% and 5%, although this capability decreases with time because of sensor drift. As a consequence, sensors modify their responses to the tested compounds compared to the responses obtained during instrument calibration.

De Vito *et al.* [89], in their study for the detection and quantification of benzene in an outdoor environment by means of an electronic nose, also encountered the same difficulty. With an electronic nose using a KNN recognition system, benzene concentration in the atmosphere was quantified with errors ranging from 2% to 6%. This error was observed to increase over time as a consequence of sensor drift.

A possible solution to the problem of sensor response drift over time is proposed by Cho *et al.* [90], who developed specific software for this purpose. Their study involved the use of an electronic nose for the detection and quantification of mixes of ammonia and hydrogen sulfide in air at different concentrations (comprised between 40 and 200 ppm for ammonia, and between 0.3 and 0.5 ppm for hydrogen sulfide). The different mixes being tested were recognized correctly at 100%, and quantification gave satisfying results, as well. Likewise satisfying results are reported by Gao *et al.* [91] who used an electronic nose with 16 sensors to analyze mixes of different VOCs at concentrations comprised between 1 and 1000 ppm.

Another set of studies that is worth to be mentioned in the field of electronic noses for the detection and quantification of specific compounds in ambient air concerns the development and the use of mobile robots [92]. Mobile olfactory robots are a particularly interesting technology in application areas where a better understanding of a gas distribution is needed.

Concerning the evaluation of gas distributions, a recent attempt to integrate gas distribution mapping together with odour classification is reported by Loutfi *et al.* [93] using a robot equipped with two electronic noses based on Figaro technology. The results of the indoor experiment proved the system to be effective in classifying the tested odorants (*i.e.*, ethanol and acetone) with classification performances between 85% and 100% depending on the distance from the source. Finally, the paper points out the importance of instrument training and the difficulties connected to the classification of odorant mixtures. In a similar paper, the authors perform a comparative study of different methods for feature extraction and explore a relevance vector machine classifier in order to further evaluate the classification performance based on the quality of the analysed samples [94].

One of the major disadvantages of the use of MOS technology for mobile robotic olfaction is that MOS sensors need long recovery period after each gas exposure. As pointed out by Gonzalez-Jimenez *et al.*, in a recent study [95], allowing for sensor recovery would force the robot to move at very low speed, almost incompatible with any practical operation. In this very interesting study the authors describe a new electronic nose that partially overcomes such limitation by comprising several identical sets of MOS sensors accommodated in four separate chambers. Good distinction of different gas (in this case, acetone) sources where obtained in several multiple gas source experiments.

In general, the studies conducted up to now regarding the use of electronic noses for the detection and quantification of specific compounds in air have generally produced promising results, even though they highlight common problems as sensor drift over time, and sensor sensitivity to interfering substances and to humidity and temperature changes. However, said studies often refer to rather high concentration of the target compounds. Future studies should focus on the development of more sensitive instruments, capable of detecting the presence of the studied gases at lower concentration levels (ppb).

As a further consideration, even though the use of electronic noses for the detection and quantification of specific gases can be very interesting for particular applications, as for instance mobile robotic olfaction, and in some cases quicker and cheaper than more traditional chemical analysis, it is also true that, the type of information obtained by the electronic nose does not represent a great novelty compared to chemical analysis. For this reason, probably the most interesting and promising applications of electronic noses for environmental monitoring might concern those fields where they are not just an alternative to chemical analyses, but where they effectively provide new and different information relevant to the analyzed gas.

#### 3.1.3. Odor Detection

As mentioned above, the most interesting applications of electronic noses regard those fields where these instruments may provide information that is not overlapping with chemical analyses. This is typically the case of electronic noses used for odor detection. As already discussed previously in this paper and in other research studies, chemical analysis involving the identification and quantification of the components of an odorant mix is not always suitable to evaluate the mixture olfactory properties [85]. For this reason, in the field of odor monitoring electronic noses might represent not just an alternative to chemical analysis, but actually the only method available to get continuous, quick and reliable information about the presence of odors in ambient air.

More in detail, in the field of environmental odor monitoring, electronic noses can be applied for two different purposes: odor quantification and/or classification. The specificities associated with both these applications are discussed below.

##### Odor Quantification

Dynamic olfactometry is the official and standardized method to quantify odor concentration in terms of odor units per cubic meter [34]. However, this method, which involves sample collection in the field and subsequent lab analysis by means of a panel of selected assessors, is not applicable for continuous monitoring purposes, because of the intrinsic discontinuity of sampling and of sensorial analysis [32]. For this reason, in the last years, several research studies have been conducted in order to evaluate the possibility to use electronic noses for such applications, with the aim of verifying reliability and repeatability of the instrument responses.

One of the first studies in this field is reported by Misselbrok *et al.* [96], in which the reliability of two different electronic noses, the Odourmapper (developed at the University of Manchester Institute of Science and Technology—UMIST) and the Aromascan PLC (Odournet, Crewe, UK), for quantifying odors was evaluated. The responses of said two instruments turned out to be linear relative to the odor concentration of the analyzed samples. However, variance of results was rather high, being equal to 59% and 62% for Aromascan and Odourmapper, respectively.

A better correlation was obtained by Stuetz *et al.* [11]. In this case, the authors, using a Neotronic Nose (Neotronics Scientific Ltd., Chelmsford, UK) with 12 CP sensors, analyzed samples collected on different wastewater treatment plant with known odor concentration, previously measured by dynamic olfactometry. By analyzing the data relevant to all the studied plants it was not possible to establish a direct correlation between odor concentrations and electronic nose responses. Instead, if analyzing the data relevant to the samples collected in each plant separately, a better correlation was obtained, covering an odor concentration range between 125 and 780,000 ou_E_/m^3^.

In order to evaluate the correlation between electronic nose responses and odor concentration, Fuchs *et al.* [42] analyzed the sensor response trends over time to verify if these were compatible with the expected odor concentration trends. After a set of preliminary laboratory tests, the electronic nose was installed inside a duck breeding farm. The sensor response trends relevant to the continuous air analysis turned out to be variable, in function of the daily animal activity cycle. This result indicates a good correlation between electronic nose responses and odor concentration. However, the study did not involve odor concentration estimation.

A similar operation was carried out in a study by Sohn *et al.* [97] who, using an Aromascan electronic nose, analyzed odor samples having odor concentrations ranging between 10 and 100 ou_E_/m^3^ coming from piggery effluent ponds. The sensor responses and the odor concentration values of the analyzed samples were used to train an artificial neural network. Based on an analysis of the raw data, the authors obtained a correlation between the real odor concentration values and the odor concentration estimated by the system having a correlation coefficient R of 0.895. Because said result was not considered satisfactory enough, the ANN was modified. This way the correlation coefficient of the new correlation turned out to be equal to 0.984. This study therefore proved how it is possible to increase the electronic nose capability of correctly quantifying odor concentration by opportunely modifying the pattern recognition system.

However, the capability to quantify odors turns out to depend, besides on the type of algorithm applied for pattern recognition, also on the range of odor concentrations to be quantified. As an example, Micone and Guy [33], using an electronic nose with 16 MOS sensors, obtained a good correspondence between estimated and real odor concentrations in a concentration range between 50 and 150 ou_E_/m^3^, these being odor concentrations that are typically encountered in ambient air at receptors.

Also, Pan and Yang [98] obtained a satisfactory correlation between estimated and real odor concentrations of samples having low odor concentrations by using an ANN, with a correlation coefficient R equal to 0.932.

The above-mentioned studies, which all show rather good results in terms of correlation between sensor responses and odor concentration, have in common that the analyses of the samples collected in the field were then carried out in the laboratory. The use of electronic noses directly in the field is quite more problematic, mainly because of the variability of the environmental conditions, such as atmospheric humidity and temperature, which negatively affect the instrument functioning. For this reason, field instruments shall be capable to minimize the effects of such variations, in order to produce reliable results. Moreover, it is fundamental to have sensors having high sensitivity to the compounds responsible for odors, because in ambient air such compounds may be present at very low concentrations (ppm-ppb) [99].

Despite the above-mentioned critical aspects, the application of electronic noses in the field for air quality monitoring at receptors, *i.e.*, directly where the odor nuisance is lamented, is nowadays of great interest. This is the reason why several studies have been conducted specifically for this purpose.

For instance, in order to evaluate the possibility to use electronic noses directly in the field to quantify odor concentration, Sohn *et al.* [73] have carried out a measurement campaign at a poultry livestock by means of dynamic olfactometry and an electronic nose equipped with 24 MOS sensors. In order to keep the instrument at levels of humidity and temperature as constant as possible, the electronic nose was installed in the field using a mobile laboratory. By comparing the concentration values measured by dynamic olfctometry and electronic nose on samples having odor concentrations comprised between 250 and 4500 ou_E_/m^3^, a linear correlation was observed, with a correlation coefficient R equal to 0.89.

Also Nicolas *et al.* [100] developed an electronic nose and then tried to use it in the field. Their study involved the verification of the instrument capability to discriminate different odors from a composting plant. More in detail, the instrument was used to evaluate the air odor concentration, in a range comprised between 500 and 20,000 ou_E_/m^3^. This study highlights that the electronic nose is capable to detect odor concentration variations, although no data validation was presented.

Another important application for an electronic nose capable to evaluate odor concentration is to combine it with odor dispersion modelling. One interesting study by Romain *et al.* [101] describes the realization of an odor dispersion model starting from the real-time data obtained by an electronic nose. However, as the correlation between estimated and real odor concentration obtained in their study was not satisfactory, they concluded that the studied instrument was unsuitable for the proposed application.

##### Odor Classification

As far as the use of electronic noses for odor classification is concerned, one of the first studies that involved the use of an electronic nose in the environmental field to assign the analyzed odor samples to a specific olfactory class was reported by Nicolas *et al.*, in 2000 [60]. After having trained the instrument with samples coming from industrial sites on different days, and with different climatic conditions, the authors have used the electronic nose in the field in order both to detect the presence of odors and to classify them, thereby using the olfactory classes identified during the training phase. Even though the instrument turned out to be able to distinguish between the different types of odors, the authors have highlighted the influence of the atmospheric conditions on the sensor responses and thus the necessity to carry out repeated training over time in order to reduce the problem of sensor drift.

Later, in 2007, Sironi *et al.* [41] have carried out an experiment in order to verify the capability of an electronic nose to classify the odorous air analyzed continuously at a receptor, exposed to the odor emissions from a composting plant. For this purpose, two electronic noses, each equipped with six MOS sensors, were specifically set up and trained with samples collected at the monitored composting plant. Then, one instrument was installed inside the plant, while the second instrument was installed in the surroundings, more precisely at a receptor located at about 4.3 km from the composting plant. The electronic noses have analyzed the air every 12 min for a 4-day period. The sensor response trends were then analyzed by means of a KNN algorithm, in order to classify the analyzed air into the different olfactory classes considered for training. The results obtained with the electronic nose installed at the receptor were compared with the recordings of odor episodes of the residents. The accuracy index of the measurements carried out by the electronic nose in the recognition of odor presence was calculated as the percent ratio between the number of correctly classified and the number of total measurements, and it turned out to be equal to 72%. An interesting observation concerns the fact that the electronic nose sensitivity turned to be higher than the sensitivity of human olfaction: 132 air measurements out of 480 were classified as odorous by the instrument, while in correspondence of said odor detections by the electronic nose, not always, at the receptor, odor presence was registered by the inhabitants. Moreover, even though the instrument turned out to be highly sensitive to the considered odors, the study also highlights the influence of atmospheric humidity on the sensor responses, and sensor response drift was observed, as well.

In order to establish which of the sensor response characteristics (*i.e.*, “features”) optimize the classification of the air analyzed in the field, Sironi *et al.* [102] carried out another field study aiming to monitor continuously the presence of odors from a composting plant. Based on the analysis of the sensor responses over time, it was possible to highlight the presence of anomalous responses due to the high humidity content of the analyzed air. By carrying out a classification of the odorous air analyzed by the instrument using different features, different results were obtained, with different accuracy indexes, ranging from 60.7% to 96.4%. By evaluating the variation of the accuracy indexes in function of the adopted features, it was possible to optimize the feature extraction in order to maximize the classification accuracy. After said optimization procedure, an accuracy of 96.4% was achieved. This study proves how the choices relevant to training and feature selection are fundamental in order to maximize classification accuracy.

A similar dependence of results towards training conditions is reported by Sohn *et al.* [35]. In this case, the electronic nose was trained with samples coming from a slaughterhouse and a pig livestock farm, with the aim of using an electronic nose for identifying the provenance of the odor detected in the analyzed air. Different accuracies were obtained by varying the number of olfactory classes being considered. More in detail, by reducing the number of olfactory classes used for training, the instrument accuracy increases up to 85%.

Besides the aspects associated with the instrument training and the data processing methods, which the above described studies prove to have substantial influence the accuracy of the recognition system, also the quality of the gas detection system is essential for the electronic nose functioning.

Sensor sensitivity towards the odor substances and the undesired cross-sensitivity to humidity and temperature variations, as well as their stability over time, are crucial aspects associated with the application of electronic noses for environmental odor monitoring.

Regarding sensor drift over time, Romain *et al.* [103] have carried out a study to evaluate its influence on the capability of an electronic nose to classify the analyzed air in a 4-year period. The experiment shows how the accuracy of classification performed by the instrument drastically decreases with time, passing from 98% to 20% at the end of the experiment. Said result highlights the necessity of carrying out repeated training over time as well as the need for an instrument capable to automatically compensate sensor drift.

More recently, in 2010, Capelli *et al.* [104] described the development of an electronic nose that, providing a daily sensor re-calibration, allows obtaining responses that are independent from sensor drift. Using this instrument, the authors have carried out a continuous monitoring in an outdoor environment, thus verifying the instrument capability to detect the presence of odors different from neutral air. Moreover, the presence of a specifically developed humidity regulator allows minimizing the influence of the humidity variations in the analyzed air.

**Figure 1.**
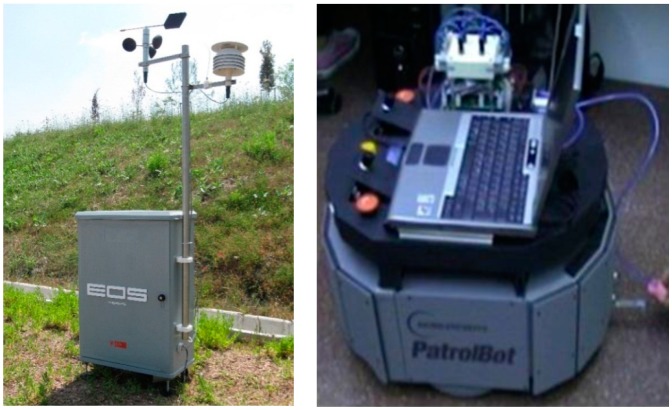
Examples of electronic noses: on the left, the EOS electronic nose by Dentoni *et al.* [105] for outdoor monitoring and, on the right, the mobile MCE-nose by Gonzalez-Jimenez *et al.* [95].

The same instrument was later (in 2012) used by Dentoni *et al.* [105] with the aim of quantifying odor. For this purpose, laboratory tests were carried out using pure compounds (limonene and ethanol) at different concentrations, and the instrument turned out to be able to correctly quantify odor of said mixes, with a square correlation coefficient *R*^2^ of 0.992 and 0.997 for limonene and ethanol, respectively. In order to evaluate the instrument's performances in the field, the study also provided the execution of an air quality monitoring, aiming to attribute the analyzed air to a specific olfactory class, by means of five electronic noses installed at receptors. The results of said monitoring campaign proved the instruments able to classify the air analyzed continuously at receptors with high accuracy. Figure 1 provides an example of this electronic nose for outdoor monitoring, together with a mobile nose.

Another continuous monitoring of air quality was carried out by Abdullah *et al.* [106]. By means of an electronic nose network, they carried out an air quality monitoring at a poultry house. The study involved the use of two electronic noses, equipped with MOS sensors, which were moved at regular time intervals to six different positions inside the poultry house. The sensor response data were analyzed by PCA in order to visualize the clustering of the measurements. The analysis highlighted how the measures relevant to a given position inside the poultry house are located close to each other in the two-dimensional space of the PCA, thus producing data clustering depending on the sampling point. Then data were analyzed using an ANN to predict odor concentration, by discriminating samples between high, medium or low concentration levels. The obtained results are promising, as the instrument was able to predict odor concentrations that turned out to be coherent with those measured at the different sampling points.

In general, these studies are all very interesting, because they prove the possibility to apply electronic noses to monitor environmental odors. The novelty of the approach is that such instruments do not perform a chemical analysis of the analyzed air, *i.e.*, they do not identify nor quantify single compounds, but they are able to detect and recognize “odor” as a whole.

### 3.2. Water Quality Monitoring

Another interesting field of application for electronic noses concerns the possibility to use them not only for air quality monitoring purposes, but also to analyze the quality of water. Traditional analytical methods are often not suitable for continuous water quality monitoring, and in many cases they turn out to be costly and entail very long times for analysis (up to some days) [107].

For this reason, electronic noses have been considered as a possible solution to satisfy the growing request for real-time indications on water quality. Such instruments, even though they never get into contact with the water, may give information about some characteristics that affect the water quality (e.g., composition, contents of specific pollutants, pH) by analyzing the air over the water surface (headspace). The studies conducted in this field up to now are mainly based on the principle of the headspace analysis.

One of the most important legislative limits fixed for wastewater treatment plants is the so called “Biological Oxygen Demand” (BOD) concentration. The BOD is a parameter that describes the degree of water pollution, and it represents the difference between the quantity of dissolved oxygen in a water sample before and after an incubation period of 5 days, at 20 °C, in presence of a bacteria flora. The analysis of the BOD therefore indicates the contents of organic biodegradable matter in wastewaters, expressed in terms of quantity of oxygen needed for the aerobic biodegradation by microorganisms [108]. For this reason, the first studies regarding the application of electronic noses for water quality monitoring focused on the measurement of this parameter.

A first study in this field was carried out by Stuetz *et al.* [26]. The existence of a correlation between the response of 12 CP sensors and the BOD content of the analyzed waters was verified. For this purpose, water samples were collected from three different wastewater treatment plants for an 8-month period. Water samples were analyzed in order to determine their BOD content, and they were subsequently placed in a closed container with neutral air. The headspace in said container was then analyzed by electronic nose. The data relevant to the BOD content and the sensor responses relevant to the different gas samples were processed using specific statistical methods such as discrimination and canonic correlation [109,110] to evaluate the correlation between the dependent variable (BOD) and the sensor response set (independent variable). Said correlation turned out to be poor if applied to the samples coming from the three plants, presumably due to the different compositions of the wastewaters. However, also by analyzing separately the data relevant to the wastewaters of each plant, the correlation between BOD and sensor response turned out to be not satisfactory, due to the fact that wastewater composition is highly variable if a large time span is considered. On the other hand, a good correlation was observed if analyzing the data relevant to a single plant and limiting the time span to a shorter period (1 month).

Regarding the correlation between BOD and electronic nose responses in function of the different periods being considered, a similar study is reported always by Stuetz *et al.* [111]. The authors used an electronic nose similar to the one described in the previously mentioned study, together with an ANN, and the aim of the study was to relate the sensor responses to the BOD values relevant to samples coming from different wastewater treatment plants, collected within a time span of 5 months. Also in this case, data correlation turned out to be rather poor, giving a correlation coefficient between BOD and electronic nose responses of 0.41. Instead, by using the same data subdivided into the month of pertinence, the canonic correlation coefficients reached values between 0.78 and 0.99. Such results prove the possibility to use an electronic nose for BOD measurements, if the instrument is periodically (e.g., every 4 weeks) re-calibrated, in order to avoid that it is excessively affected by the variations that typically occur in wastewaters. Moreover, the fact of carrying out repeated training of the instrument over time reduces the risk of sensor responses to be affected by sensor drift.

In order to remedy to such problems without needing to re-calibrate the instrument every month, Dewettinck *et al.* [12] proposed to carry out data pre-processing, by normalizing them relative to the sensor resistivity value obtained in the same day by flushing a known reference substance onto the sensors. More in detail, in order to establish a correlation between sensor responses and the presence of volatile compounds in the water samples, for each analysis, and for each sensor, the difference between the resistivity registered during the analysis and the resistivity relevant to the base line was calculated. Said resistivity value was named by the authors as “sensorial odor perception” (SOP). Then, in order to evaluate the correlation between the different SOP values relevant to the analysis of the 21 water samples and the VOC concentration, the SOP values of the samples were divided by the SOP measured for the reference substance in the same day. Thanks to this data processing procedure, repeatable results were obtained. Nonetheless, the system turned out to be able to discriminate the different samples containing volatile compounds only if the liquid samples were heated, because at room temperature the volatilization of the organic compounds contained in the liquid phase was not sufficient to make it detectable by the electronic nose. More in detail, a good discrimination between the waters containing VOCs and those not was achieved by increasing the heating temperature up to 90 °C. Said result, although satisfactory, makes the developed system hardly applicable to a continuous water quality monitoring, due to the difficulties associated with the need to carry out a continuous withdrawal and heating of the water sample before headspace analysis.

On the other hand, an application that provides to directly analyze the air over the water surface without any liquid withdrawal is reported by Lamagna *et al.* [112], who used a commercial electronic nose (Cyrano320 nose, Cyrano Sciences Inc., Hertz, UK) to evaluate the presence of pollutant compounds into the river Riachuelo in Argentina, by analyzing the air at about 1 m over the liquid surface. The study allowed the authors to establish the correlation between the sulfides and nitrates concentration in the water and the electronic nose responses by means of canonic correlation analysis. Moreover, a similar correlation was observed between the concentration of heavy metals in the water and electronic nose responses. The study indicates the possibility to use an electronic nose to detect the presence of non-allowed discharges of effluents in superficial waters.

A study by Baby *et al.* [113] had a similar aim, *i.e.*, to use an electronic nose in order to verify the possibility of detecting the presence of pollutants and pesticides in superficial waters, and to determine their detection limits. This study, conducted using an electronic nose equipped with both MOS and QCM sensors, reports a linear correlation between lindane concentration and sensor response values along the first principal component obtained by PCA. The detection limit for lindane turned out to be 1 ppm, whereas for aqueous samples containing nitrobenzene it was 500 ppm. Finally, the study proved the electronic nose to be able to recognize the presence of pesticides and to discriminate them.

A more recent application of electronic noses in water quality monitoring concerns the detection of microorganisms. The study by Bastos and Magan [44] aims to identify the presence of microorganisms responsible for the emission of odorous substances in waters. More in detail, in order to prevent the microorganisms to make the water odorous and thus unpleasant, the detection of the presence of such microorganisms shall be carried out in a short time interval. For this purpose, an electronic nose equipped with 14 CP sensors was used, which analyzed the headspace over the water samples inoculated with the microorganisms. Based on specific laboratory testing, the instrument turned out to be able to detect the presence of *Streptomyces* after only a 24-h growth. Moreover, the instrument was also able to discriminate non-contaminated water from water contaminated by *Streptomyces* at a concentration of 10^2^ spores/mL by means of PCA.

### 3.3. Process Control

The studies described in the previous paragraphs prove that electronic noses are suitable for the verification of the contamination of air or water. However, said instrument also may be useful as prevention tools: electronic noses can be applied to process control, in order to promptly detect the occurrence of anomalies and thus prevent contamination.

The studies that were carried out up to now in this field were mainly focused on the use of electronic noses applied to two specific processes, which are composting and wastewater treatment. Moreover, laboratory experiments were carried out in order to evaluate the possibility to use electronic noses to monitor the processes that occur inside bio-reactors and anaerobic digesters.

Regarding the possibility to monitor the composting process, the studies conducted up to now focus on two main applications: the control of compost maturation and the onset of undesired anaerobic degradation processes. Both applications are useful to ensure a good process trend, the quality of the final product (compost), as well as a minimized impact on the surroundings, in terms of VOC and odor emissions.

The studies regarding the possibility to use electronic noses in order to determine the degree of compost maturation were conducted in laboratory, both by re-creating at hoc composting mixtures or by using compost samples collected directly on real plants.

As an example, Lieberzeit *et al.* [54] have carried out a laboratory study using pine needles and grass together with a composting accelerator placed inside a composting bin. The exhaust air from the bin was analyzed by means of GC-MS and an experimental electronic nose equipped with six QCM sensors. Given that as the composting process proceeds, different substances are released into the air, it was possible to establish a correlation between the results of the chemical analyses of the air and electronic nose responses, thus making it possible to subsequently apply the instrument for the continuous monitoring of the compost maturation process. The concentrations of some key components estimated by the electronic nose, such as ethyl acetate, alcohols and terpenes, turned out to be comparable with those measured by gas chromatography. Based on said correspondences, the tested electronic nose turned out to be capable, once opportunely calibrated, to partially replace classical analytical methods, guaranteeing both good sensitivity and reproducibility, thus allowing to carry out a continuous monitoring of the composting process.

A similar approach was used by Romain *et al.* [31], who verified the possibility to use an experimental electronic nose equipped with seven MOS sensors with the aim to analyze the exhaust air above a compost heap, and to monitor both the maturation degree and the onset of undesired anaerobic conditions. Data relevant to the chemical composition of the analyzed air (obtained by GC-MS) were related to the sensor responses, thereby obtaining a good correlation. However, the study highlights that the variations of temperature and humidity of the analyzed samples significantly affect the sensor responses. This phenomenon proves the necessity to carry out further studies in order to make the instrument more robust and reliable towards temperature and humidity variations of the analyzed samples.

An analogous application of electronic noses for composting process monitoring is reported by Figueiredo and Stentiford [30], who used an electronic nose to detect the transition of the composting process from aerobic to anaerobic. Using a laboratory reactor with controlled temperature, besides analyzing the air with an electronic nose developed by Bloodhound Sensors Ltd. (Normanton, UK), the authors also monitored different parameters over time: redox potential and oxygen concentration. Moreover, odor concentration was measured by dynamic olfactometry. The reactor was kept closed, so that the oxygen concentration inside the reactor progressively decreased to a level below which the process turned from aerobic to anaerobic. The transition phase was monitored by means of the electronic nose as well as by analyzing the data from the other measurements. The electronic nose turned out to be able to identify this change by producing different responses in function of the ongoing process. This study also highlights a problem associated with sensitivity of the utilized sensors toward temperature variations. The same problem was noticed also in the application of electronic noses to wastewater treatment process control.

Bouregois and Stuetz [114] have pointed out that the sensors responses of the electronic nose they used, equipped with 12 CP sensors, were influenced by humidity and temperature variations in the analyzed samples, which were withdrawn from the headspace of a chamber containing waters coming from a wastewater treatment plant. Despite that important limitation, the study, which aimed to evaluate if the electronic nose was capable of discriminating between water samples belonging to different treatment phases and coming from different wastewater treatment plants, gave satisfactory results. More in detail, the measures relevant to waters belonging to different treatment phases were analyzed by PCA, and they turned out to be clearly discriminated along the first principal component, which expressed about 93% of the total variance of the acquired data. On the other hand, the second principal component, which expressed only 5% of the original data total variance, which is not useful for the discrimination of the samples, turned out to be representative of the variability of the electronic nose responses towards similar samples having different temperatures and different humidity contents. However, the study did not involve a verification of the quality of classification.

Cross validation was applied in order to verify the quality of classification in a study by Lozano *et al.* [62], who used an electronic nose with MOS sensors to analyze polluted water samples either appositely realized in the laboratory or collected from a real wastewater treatment plant. The electronic nose was used to run headspace measurements, and data were then analyzed by means of PCA. The data relevant to the pollutant waters realized in the laboratory turned out to be well discriminated in function of the pollutant compound added to the water. Moreover, the system was able to classify the analyzed samples in function of the contained pollutant by means of Radial Basis Function Networks (RBFs) [115], thus obtaining 100% of correct classifications relevant to samples prepared in the laboratory. The system also correctly classified 90% of the samples coming from the real wastewater treatment plant. The studied electronic nose thus turned out to be suitable for monitoring water quality in wastewater treatment plants, as it was able to discriminate to a satisfactory level water samples coming from different plant sections, characterized by different pollutant loads.

Another approach adopted in different studies involves the estimation of the pollutant load of the analyzed waters, without distinguishing said waters based on their wastewater treatment process of provenance. Bourgeois *et al.* [43] used an electronic nose equipped with 12 CP sensors for the continuous monitoring of variations in wastewater quality at the plant located at the Cranfield University. The sensor responses relevant to the analyzed samples turned out to be compatible with the cyclic variations of wastewaters quality. Moreover, in order to evaluate the instrument capability to detect the presence of different pollutants, different concentrations of diesel fuel (0.2% *v*/*v* and 0.4% *v*/*v*) were added to the analyzed waters, which were immediately detected by the sensors. An innovative aspect of this study is that the authors developed a specific mathematical model, based on the comparison of the sensor responses to a variable mean value, estimated by said model, in order to minimize the influence of sensor drift on the system capability to detect the presence of pollutant compounds.

Another study aiming to characterize and monitor the quality of polluted waters was conducted by Sohn *et al.* [116]. The work had the aim to use electronic noses for the characterization of pollutant waters in terms of volatile solids load, by correlating said parameter with the odor concentration of the air over the polluted water (headspace). This study has shown that, even though the volatile solids contents is linearly correlated with other physical properties of the liquid, said correlation is not valid for odor concentration, which doesn't have a linear trend with the volatile solids contents. Nonetheless, after suitable training carried out with samples at known odor concentration, measured by dynamic olfactometry, the electronic nose was able to estimate the odor concentration of the air in contact with the polluted water. By means of an ANN, a correlation index *R*^2^ of 0.98 was achieved between estimated and real odor concentration values.

Recent developments in this field aim to develop an instrument that is as less as possible affected by atmospheric conditions and by humidity and temperature variations that typically occur during monitoring.

Regarding the use of electronic noses for process control in anaerobic digesters, Gilles *et al.* [117] have carried out a laboratory study in order to evaluate the applicability of the instruments to detect variations in the organic load. The study involved the use of an electronic nose equipped with six MOS sensors, and the reactor was simulated using a closed container having a 1.8 L-volume, fed with organic matter. The feeding was varied over time, and the results prove that the MOS sensor responses vary according to the organic contents, thus allowing to identify possible overloading situations.

The application of electronic noses for process control in bio-reactors was discussed in a preliminary study by Rosi *et al.* [118]. The experiment involved the use of an electronic nose for the continuous analysis of the air inside the bio-reactor, above the liquid where the reaction takes place. By applying PCA to the sensor responses, a clustering of said responses over time is obtained, which depends on the ongoing reaction phase. In fact, the electronic nose is able to discriminate the bacteria inoculation phase inside the reactor from the successive acclimation phase and the phase when the bio-reaction reaches the maximum velocity. This study proves that electronic noses could be useful to monitor bio-reactors, guaranteeing the continuous control of the ongoing process. Further studies will however be required in order to verify the applicability of said instruments to the monitoring of a wide range of different bio-reactors, operating at different conditions. In general, the application of electronic noses to process control turns out to be promising, especially because of the possibility to carry out continuous monitoring over prolonged time spans. Further studies will be required to fine-tune the instruments, in order to obtain reliable and stable responses over time.

### 3.4. Efficiency Verification of Odor and Pollutant Abatement Systems

Many industrial activities adopt systems for the abatement both of pollutant substances and of odors in the emissions, in order to control the environmental impacts. Such systems typically include scrubbers or biofilters, installed upstream before emission into the atmosphere of the treated gas stream. The efficiency of said systems shall be periodically verified, in order to, if necessary, modify the systems operational parameters as to guarantee emission abatement. Efficiency verifications may be carried out discontinuously, by periodically analyzing odor or pollutant concentrations, or continuously, using specific sensors to detect the presence of odor or pollutant compounds in the abatement systems outward flow. Electronic noses can be applied in this field in order to characterize emissions, as replacement or integration of specific sensors or of olfactometric measurements.

Few studies regarding this kind of applications were published up to now, due to the high specificity of the application. The main critical aspect that has limited research in this field is the instrument requirement to estimate odor concentration in a sufficiently accurate manner. In fact, in order to develop an instrument suitable for the monitoring of odor abatement systems, it is important to verify the capability of said instrument to detect odor concentration variations that may occur due to the monitored system's malfunctioning. Another critical aspect is the high humidity contents that is typically encountered in gas streams coming out from abatement systems (especially scrubbers and biofilters).

A first study regarding the possibility to use an electronic nose to detect odor abatement systems failure was carried out by Stuetz and Bourgeois [119], who conducted a continuous monitoring of the headspace of a specific system containing waters withdrawn from a wastewater treatment plant. The electronic nose they used for the study turned out to be able to detect variations in the air odor concentration, and is therefore considered suitable as detector of possible anomalies downstream of odor abatement systems in wastewater treatment plants. A first study on a real abatement system was reported by Sohn *et al.* [116]. More in detail, the authors investigated the possibility to monitor a biofiltrating system for the treatment of odorous air from a pig livestock, using a commercial electronic nose, Aroma Scan A32S, equipped with 32 CP sensors. The data registered by the instrument were pre-processed using a specific sensor calibration algorithm to compensate responses to humidity, and then analyzed with three different techniques, in order to evaluate the best performing in terms of odor concentration determination. At the same time, 81 air samples were collected for olfactometric analysis, in order to have odor samples at known concentration for the instrument training. By using a linear regression based on the sensor responses, and considering the first three principal components, the correlation between the odor concentration values measured by dynamic olfactometry and those estimated by the electronic nose gave a correlation coefficient *R*^2^ of 0.44. Such an unsatisfactory value may be due to the fact that the sensor responses do not vary linearly with the odor concentration, thus a linear model is unsuitable for this purpose. As a consequence, two different models were tested, by using an ANN and a PLS. The two methods produced better results than those obtained by PCA, with *R*^2^ values equal to 0.62 and 0.79, respectively. Even though the result obtained by using the ANN is negatively affected by the relatively low number of samples with which the system was trained (81), the regression based on PLS turned out to be the best method for odor quantification (but not for odor classification).

These results, although very promising, are to be considered as just a first step towards a reliable application of electronic noses for the efficiency verification of odor and pollutant abatement systems. As a matter of fact, given the high variability of the gas composition at the outlet of different treatment systems and the high degree of complexity required for electronic noses to be used for this purpose, said instruments need to be fine-tuned specifically for the different industrial activities, as to guarantee system accuracy and robustness.

## 4. Conclusions

Electronic noses are an interesting and promising technology in the environmental field, both for odor impact assessment [120,121] or control [119] application purposes. Once opportunely trained, electronic noses can be used successfully for both detecting and identifying odors, by attributing the analyzed air to an olfactory class corresponding to a specific odor source. With respect to other measurement methods involving the use of human assessors, instrumental analysis with electronic noses entails the great advantage of allowing the measurements to be run continuously [105], and at lower costs. The studies conducted in order to evaluate the possibility to use electronic noses in the environmental field have proved that said instruments are generally suitable for the different applications reported, if the instruments are specifically developed and fine-tuned. As a general rule, literature studies also discuss the critical aspects connected with the different possible uses, as well as research regarding the development of effective solutions for said problems. Regarding the sensors, several studies have highlighted the problem of stability towards temperature and humidity variations, as well as sensor response drift over time. As an example, a recent study by Dentoni *et al.* [105] describes an innovative electronic nose, specifically developed for environmental monitoring, which includes specific equipment to compensate sensor drift and to regulate sample humidity. This is just an example to prove that, in general, as discussed in most research works cited in this paper, electronic noses for environmental monitoring applications are not trivial. On the contrary, they require sophisticated and complex technology in order to produce accurate and reliable results. Actually, there are several extremely simple devices commercially available, which are generically defined as “electronic noses”, able for instance to detect gas leaks or evaluate single gas concentrations. It is important to highlight that such simple instruments are unsuitable for environmental monitoring purposes.

Future challenges regarding the use of electronic noses in the field of environmental monitoring shall presumably not be focused on the development of new sensors or data processing methods, but rather concentrate on the adjustment of the instrument for outdoor applications. In future, it would be extremely interesting to have electronic noses able to tolerate the variability that is typical of real environmental applications, as well as mobile electronic noses for field-inspection-like [121,122] applications.

However, the most critical aspect actually limiting the use of electronic noses for environmental applications is the lack of specific regulation for their standardization. As previously mentioned, electronic noses for environmental monitoring are extremely complex instruments, and their use entails a large number of degrees of freedom, regarding for instance the training and the data processing procedures. Actually, the definition and standardization both of the instruments characteristics and performances, and of the procedures for their correct utilization is a necessary requirement for their diffusion. One first standardization attempt, fixing how to apply electronic noses in environmental monitoring, is represented by the NTA-905, which is a technical agreement document released in December 2012 by the Netherlands Standardization Institute [123].

## Figures and Tables

**Table 1.** Sensor types used in electronic nose systems.

**Type**	**Sensitive Material**	**Varying Property**
Membrane-oxide semiconductor field-effect transistor (MOSFET)	Semiconductor layer + layer of catalytic metals	Conductivity/electric field
Colorimetric sensors	Organic dyes	Color, absorbance
Electrochemical sensors	Solid or liquid electrlytes	Current/voltage
Fluorescence sensors	Fluorescence-sensitive detector	Fluorescent light emissions
Infrared sensors	IR-sensitive detector	Infrared-radiation absorption
Optical sensors	Photodiode, light-sensitive	Light modulations
